# Network dysfunction underlying verbal fluency deficits in newly diagnosed epilepsy: a resting-state fMRI functional connectivity study

**DOI:** 10.1186/s12916-025-04577-y

**Published:** 2025-12-13

**Authors:** Fangzhou Liu, Ping Jiang, Ge Tan, Xiuli Li, Deng Chen, Yingchun Xu, Zixian Zhou, Qiyong Gong, Ling Liu

**Affiliations:** 1 Department of Neurology, West China Hospital of Sichuan University, 37# Guoxue Lane, Chengdu, Sichuan 610041 China; 2 Department of Radiology, Huaxi MR Research Center (HMRRC), Institution of Radiology and Medical Imaging, West China Hospital of Sichuan University, Chengdu, Sichuan China; 3 West China Medical Publishers, West China Hospital, Sichuan University, Chengdu, 610041 China; 4 Psychoradiology Key Laboratory of Sichuan Province, West China Hospital of Sichuan University, Chengdu, Sichuan China; 5 Mental Health Center, West China Hospital of Sichuan University, 37# Guoxue Lane, Chengdu, Sichuan 610041 China; 6 Xiamen Key Lab of Psychoradiology and Neuromodulation, Department of Radiology, West China , Xiamen Hospital of Sichuan University, Xiamen, Fujian China

**Keywords:** Newly diagnosed epilepsy, Verbal fluency test, Resting-state fMRI, Independent component analysis, Functional connectivity

## Abstract

**Background:**

Verbal fluency impairment is a common cognitive deficit in epilepsy that further increases the burden of the disease. Current anti-seizure medications mainly target seizure control but rarely improve cognition and may even worsen it in the patients. Neuromodulation has shown potential to control seizures and improve cognition simultaneously by stimulating specific nuclei or neural circuits, but the precise targets for verbal fluency deficits remain unclear, particularly in patients unaffected by anti-seizure medication. Therefore, investigating the neural mechanisms in newly diagnosed epilepsy (NDE) patients is essential for developing targeted interventions. This study aimed to explore brain network abnormalities and their relationship with verbal fluency deficits in NDE patients.

**Methods:**

One hundred NDE patients and 54 matched healthy controls were recruited and underwent resting-state functional magnetic resonance imaging (fMRI). Independent component analysis was used to assess whole-brain network functional connectivity (FC). Verbal fluency was evaluated using character and semantic verbal fluency tests (VFTs). Correlation and mediation analyses were conducted to examine the relationships among clinical features, verbal fluency, and FC.

**Results:**

Compared with healthy controls, NDE patients exhibited hypo-intra-network FC in the medial visual, auditory, and lateral sensorimotor networks. Correlation analysis showed that FC in the left auditory network, including the left inferior frontal gyrus (IFG) and superior temporal gyrus (STG), was significantly associated with the VFT scores in the NDE patients. Notably, the FC of IFG and STG within the left auditory network mediated the relationship between seizure frequency and verbal fluency deficits.

**Conclusions:**

These findings indicate that NDE patients exhibit widespread dysfunction in perceptual networks. Abnormal FC of IFG and STG within the left auditory network serves as a neural substrate linking seizure burden to verbal fluency deficits. These insights provide a foundation for future interventions targeting network-level dysfunction in patients with epilepsy.

**Supplementary Information:**

The online version contains supplementary material available at 10.1186/s12916-025-04577-y.

## Background

Epilepsy is one of the most common neurological disorders, affecting about 70 million people and posing a major public health burden [1, 2]. Beyond recurrent seizures, over 60% of patients have cognitive impairments, which further worsen outcomes [3]. Current anti-seizure medications (ASMs) focus on seizure control, yet no effective ASMs exist for cognitive deficits, and some ASMs may even exacerbate cognitive dysfunctions. Neuromodulation is a promising alternative treatment for enhancing cognition in epilepsy. For example, deep brain stimulation has been increasingly investigated as a strategy to control seizures and improve cognitive outcomes through targeted modulation of specific nuclei or neural circuits [4]. However, the complexity of epilepsy-related brain networks makes it difficult to identify precise therapeutic targets. Therefore, investigating the neural mechanisms underlying cognitive impairment in epilepsy is essential for developing targeted and effective interventions.

Verbal fluency decline is a common cognitive impairment in epilepsy, which can adversely affect daily communication and reduce patients’ quality of life [5]. Verbal fluency is usually assessed with verbal fluency tests (VFTs), including phonemic tests (e.g., generating words beginning with a specific letter) and semantic tests (e.g., naming items within a category such as animals) [6]. Clinical studies have shown that earlier onset age, longer disease duration, and more frequent seizures are associated with more severe verbal fluency deficits [7–9]. Lesion-symptom mapping and task-based functional magnetic resonance imaging (fMRI) studies have identified specific brain regions involved in verbal fluency. Phonemic fluency mainly depends on the left frontal regions, particularly the inferior frontal gyrus (IFG), while semantic fluency relies more on the left temporal gyri [10]. Additionally, there is a consensus that both phonemic and semantic fluency are associated with the left frontotemporal network [10–13].

However, several gaps remain. First, most studies focused on chronic epilepsy, making it unclear whether verbal fluency–related brain changes arise early in the disease or later with progression or ASM effects. Certain ASMs, such as topiramate and zonisamide, can impair cognition and alter brain networks [14]. Studying newly diagnosed epilepsy (NDE) can help mitigate the confounding effects of ASMs [15–17]. Second, prior imaging studies mainly examined individual brain regions or a single network, ignoring the whole-brain network mechanisms. Because epilepsy is a network disorder, data-driven methods such as independent component analysis (ICA) can map whole-brain functional connectivity (FC) and provide a more comprehensive view of brain function. Third, most verbal fluency studies have used English-language tests, while evidence from Chinese stimuli remains limited. Because Chinese relies on characters rather than an alphabet [18], character-based VFTs (e.g., using “发” to generate words like 发现/discovery or 发明/invention) are more suitable for Chinese speakers compared with letter-based VFTs [19, 20]. Using character VFTs may help advance understanding of the linguistic mechanisms specific to Chinese populations [21].

Therefore, our study aimed to use resting-state fMRI and data-driven ICA to explore whole-brain FC abnormalities underlying verbal fluency in NDE patients compared with healthy controls (HCs). We also assessed associations between clinical features (age of onset, epilepsy duration, seizure frequency), verbal fluency performance (character and semantic VFTs), and FC through correlation and mediation analyses. We hypothesized that (a) NDE patients would show left frontotemporal network abnormalities compared to HCs, (b) these abnormalities would be associated with verbal fluency deficits, and (c) these network alterations would mediate the relationship between clinical features and the severity of verbal fluency deficits.

## Methods

### Participants

This study was approved by the Ethics Committee on Biomedical Research, West China Hospital of Sichuan University. Written informed consent was obtained from all participants. One hundred NDE patients were recruited from the outpatient and inpatient departments of West China Hospital of Sichuan University, between 2019 and 2024. All patients were diagnosed by two experienced neurologists (LL and FL) following the 2014 definition by the International League Against Epilepsy [22]. Inclusion criteria for NDE patients: (1) age between 14 and 65 years; (2) no prior use of ASMs; (3) negative visual structural MRI. As no universal definition of MRI-negative exists [23], we defined it as the absence of structural abnormalities (e.g., hippocampal sclerosis, focal cortical dysplasia, tumors) on visual inspection of T1-weighted and T2-FLAIR images, and confirmed by radiologists and an experienced neuroradiologist (GT). Exclusion criteria for NDE patients: (1) inability to undergo MRI scanning (e.g., due to claustrophobia, vascular stents, pacemakers, or dental metalwork); (2) presence of intellectual disability or dementia; (3) comorbid neurological or psychiatric disorders (e.g., encephalitis, stroke, major depression, or schizophrenia); (4) inability to communicate in Mandarin.

A total of 54 HCs, matched for age, sex, and education, were recruited through advertisements at West China Hospital of Sichuan University and included for group comparisons with NDE patients. Additionally, 100 HCs were sourced from an existing database at the Huaxi Magnetic Resonance Research Center and utilized to generate group ICA spatial templates. Inclusion criteria for HCs were as follows: (1) no prior use of neuropsychiatric medications; (2) a normal head MRI without identifiable lesions; (3) no history of neurological or psychiatric disorders. Exclusion criteria for HCs included (1) contraindications to MRI; (2) intellectual disability or dementia; and (3) inability to communicate in Mandarin.

### Demographic, clinical, and neurocognitive assessments

Demographic and clinical data were collected for all NDE patients, including (1) demographic information: gender, handedness, age at scanning, and years of education and (2) clinical characteristics: age of onset, epilepsy duration, history of febrile seizures, history of status epilepticus, family history of epilepsy, seizure frequency, seizure types, and electroencephalography (EEG) reports. For HCs, demographic data included gender, handedness, age at scanning, and years of education.

All NDE patients underwent the following assessments conducted by experienced neurologists (FL and DC): (1) global cognitive function was assessed with the Raven’s Standard Progressive Matrices (RSPM), a widely used nonverbal measure of reasoning and fluid intelligence, relatively independent of language and cultural background [24]. The test comprises 60 progressively challenging pattern-matching tasks, yielding a raw score (0–60) convertible to a percentile score. (2) Verbal fluency tests, including a character VFT (participants were instructed to generate as many words as possible beginning with the syllable “发” /fa/ within 1 min) and a semantic VFT (participants were instructed to name as many animals as possible within 1 min) test [20].

### MR data acquisition

All participants underwent MRI scanning using a 3.0 T imaging system (Tim Trio, Siemens Healthineers, Erlangen, Germany) equipped with a 12-channel phased-array head coil. High-resolution three-dimensional T1-weighted images were acquired using a spoiled gradient-recalled echo sequence, with the following parameters: repetition time = 1900 ms, echo time = 2.26 ms, flip angle = 9°, field of view = 256 × 256 mm^2^, matrix size = 256 × 256, slice thickness = 1 mm, voxel size = 1 × 1 × 1 mm^3^, and 176 axial slices. Resting-state functional images were acquired using a gradient-echo echo-planar imaging sequence to capture blood oxygen level–dependent signals, with the following parameters: repetition time = 2000 ms, echo time = 30 ms, flip angle = 90°, field of view = 240 × 240 mm^2^, matrix size = 64 × 64, slice thickness = 5 mm, voxel size = 3.75 × 3.75 × 5 mm^3^, and 30 axial slices. Each functional imaging session consisted of 200 time points for a total scanning duration of 400 s.

A pre-scan session was performed to allow participants to adapt to the environment. During scanning, participants were instructed to keep their eyes closed, remain awake, and avoid focusing on any specific thoughts. Earplugs were provided to minimize noise, and foam pads were used to reduce head motion.

### MR image preprocessing

T1-weighted data and resting-state fMRI were preprocessed using the FMRIB Software Library (FSL, version 6.0.7.12) [25]. For T1-weighted images, the Brain Extraction Tool (BET, version 2.1) was used to remove non-brain tissue, generating a skull-stripped T1-weighted image. For resting-state fMRI data, preprocessing steps included skull stripping, motion correction, slice timing correction, and spatial smoothing with a Gaussian kernel of 5 mm full width at half maximum (FWHM), all performed using the FMRI Expert Analysis Tool (FEAT, version 6.0). Functional images were first registered to individual T1-weighted images using FMRIB’s Linear Image Registration Tool (FLIRT, version 6.0) and then normalized to the MNI152 2 mm standard space using FMRIB’s Nonlinear Image Registration Tool (FNIRT, version 6.0).

To further minimize motion artifacts, Independent Component Analysis–Automatic Removal of Motion Artifacts (ICA-AROMA), a data-driven approach, was used to identify and remove residual motion-related components [26]. Specifically, probabilistic ICA decomposed the fMRI data into independent components, and a classification algorithm was applied to identify motion-related components, which were subsequently removed using the “fsl_regfilt” command [27]. Finally, temporal high-pass filtering (cutoff: 0.01 Hz) was applied to the denoised dataset. A detailed flowchart of the preprocessing is provided in Additional file 1: Fig. S1.

### Deriving functional brain networks

The denoised resting-state fMRI data from 100 HCs were analyzed using the MELODIC toolbox in FSL to create spatial templates for subsequent analyses [28]. Consistent with the method described by Lai et al. [27], Beckmann and Smith [29], and Jiang et al. [30], group-level temporal concatenation ICA was performed using a predefined number of components (i.e., 30 components). These components were then evaluated through visual inspection by experienced researchers (PJ and FL) and spatial correlation analysis using “fslcc” to compare them with established templates. Ultimately, 18 intrinsic connectivity networks (ICNs) were identified, including the visual, auditory, language, sensorimotor, default mode, frontoparietal, dorsal attention, salience, and subcortical networks (Additional file 1: Fig. S2). The network nomenclature used in this study was based on prior literature [31–33].

### Functional connectivity analysis

#### Intra-network functional connectivity

To calculate intra-network FC, the dual regression was employed [34], consisting of three stages [35]. In the first stage, individual time courses were extracted by regressing each participant’s fMRI data onto the group-level spatial templates. In the second step, these time courses were used to regress each participant’s fMRI data back onto the spatial templates, generating individual spatial maps that represent the voxel-wise FC within each ICN. In the third step, group-level analyses were conducted by comparing the individual spatial maps between the HCs and NDE patients. Age, gender, and years of education were included as covariates to control for confounding factors. The statistical significance of intra-network FC differences was assessed using FSL’s Randomize non-parametric permutation test (5000 permutations) [36]. Threshold-free cluster enhancement (TFCE) was applied for thresholding, and family-wise error (FWE) correction was used to control for multiple comparisons across the whole brain. Notably, the corrected *P* values obtained from Randomize were fully corrected for voxel-wise multiple comparisons; however, additional correction was required for multiple comparisons across all ICNs. Given the absence of strong a priori hypotheses, both increased and decreased ICN connectivity were considered meaningful. Therefore, the final statistical threshold was set at Bonferroni-corrected *P* < 0.05 / (18 × 2) = 0.001.

In addition, individual mean *z*-scores representing intra-network FC strength were extracted from each significant cluster identified in the group comparison. Each significant cluster was first binarized as a mask, and mean *z*-scores were then calculated from the dual-regression stage-2 *z*-maps for each subject. When multiple clusters were detected within the same network, separate *z*-scores were obtained and analyzed individually. These values served as quantitative measures of intra-network connectivity and were subsequently correlated with clinical and behavioral variables.

#### Between-network functional connectivity

Between-network FC was calculated using the FSLNets toolbox in Python [37]. First, the time series corresponding to each spatial template was extracted for all participants using the output from stage 1 of the dual regression process. Then, ICNs with significant group-level differences in intra-network FC were used as regions of interest (ROIs). Subsequently, partial correlations between the time series of the ROIs and other ICNs were computed using L1 regularization, resulting in an *n* × 18 connectivity matrix for each participant (*n* represents the number of ROIs). Finally, group-level comparisons of between-network FC strength were performed between HCs and NDE patients using FSL’s Randomize tool with 5000 permutations [36]. Age, gender, and years of education were included as covariates. TFCE was applied for thresholding, with results corrected for multiple comparisons (FWE *P* < 0.05).

Additionally, individual mean *z*-scores representing between-network FC strength with significant group differences were extracted for subsequent analysis. Pairwise correlations between network time series were computed for each subject using FSLNets and transformed to Fisher’s *z*-scores. For network pairs showing significant group differences, the mean *z*-scores were extracted as quantitative measures of between-network connectivity and subsequently correlated with clinical and behavioral variables. The above results were visualized using FSLeyes and MATLAB 2023b.

### Statistical analyses

#### Group comparisons

Categorical variables were described using frequencies and tested using Pearson’s chi-square test. Continuous variables were expressed as mean ± standard deviation (SD) if they followed a normal distribution and were compared using an independent-sample *t*-test. For variables not following a normal distribution, data were presented as medians with interquartile ranges and analyzed using the Mann–Whitney *U* test.

#### Correlation analyses

Correlation analyses were first conducted to explore the relationship between clinical features and VFT scores in NDE patients. Subsequently, the analyses further explored whether brain network FC strength with significant group-level differences were associated with both VFT scores and clinical features. All analyses controlled for age, gender, years of education, and global cognitive function (i.e., RSPM score). Statistical significance was determined at a two-tailed *P* < 0.05, with Bonferroni correction applied for multiple comparisons (*α* = 0.05/*n*, *n* represents the number of correlation tests performed).

#### Mediation analysis

Mediation analyses were conducted using SPSS PROCESS v4.2 (model 4) to assess whether brain network FC strength mediated the relationship between clinical features and VFT scores. This method evaluates whether the effect of an independent variable (IV) on a dependent variable (DV) is transmitted through a mediator (M) [38]. In this study, clinical features associated with VFT scores were considered as IV, brain network FC strength associated with both VFT scores and clinical features was treated as M, and VFT scores were treated as the DV. Age, gender, years of education, and global cognitive function (i.e., RSPM score) were included as covariates in the analysis. The analyses used 5000 bias-correction bootstrap samples, and statistical significance was defined as the 95% confidence interval (CI) not including zero.

#### Subgroup analysis

We conducted subgroup analyses by seizure type (focal vs. generalized) and seizure laterality. Because definitive lateralization typically requires a multidisciplinary evaluation integrating clinical semiology, ictal EEG, structural MRI, positron emission tomography–magnetic resonance imaging (PET-MRI), and postsurgical outcomes [39–41], in this study, we classified patients into possible left- or right-onset subgroups based only on clinical semiology and ictal EEG findings.

**Fig. 1:**
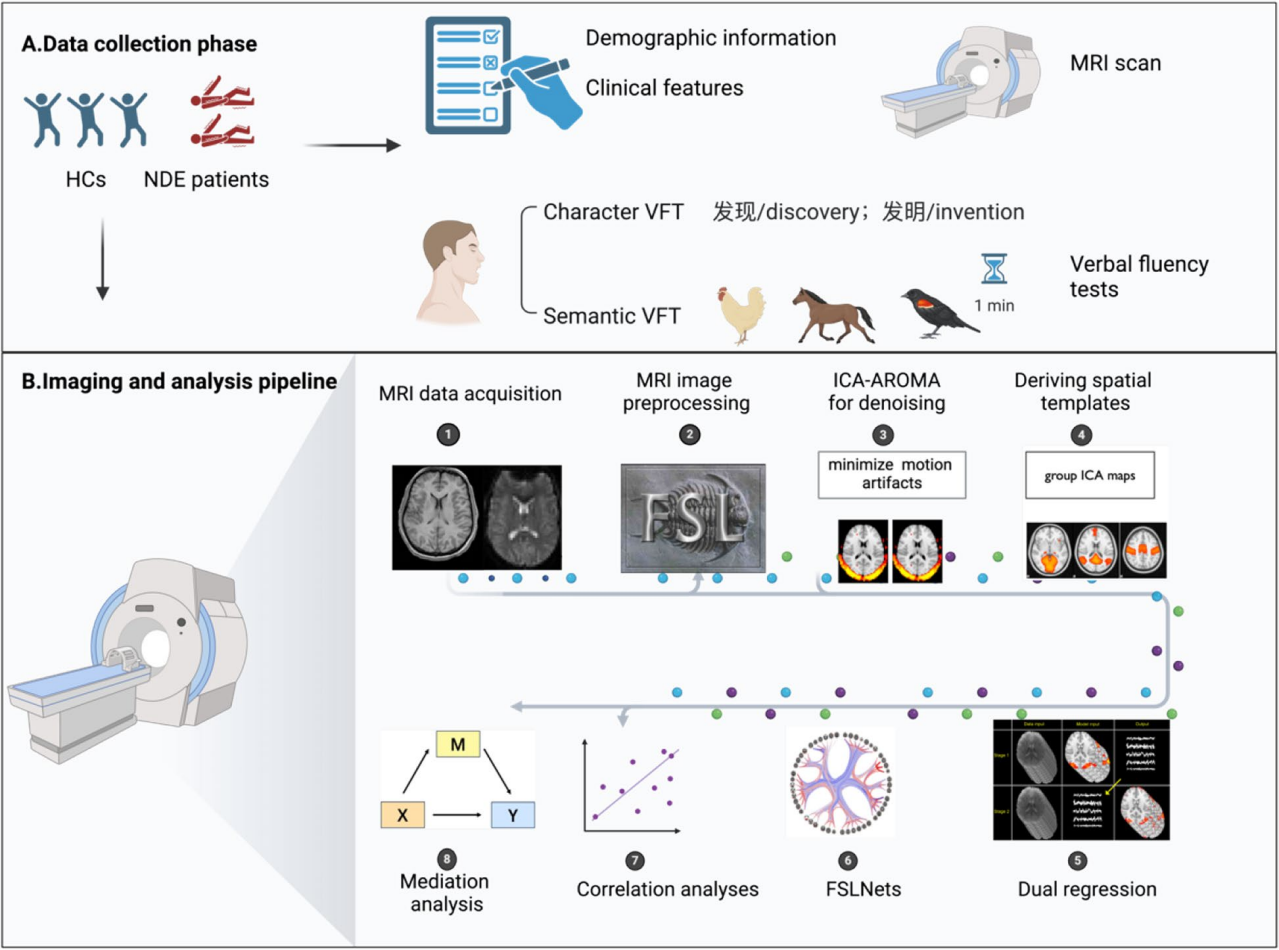
Flowchart of the study. **A** Data collection phase: HCs and NDE patients were included, and demographic information, clinical features, and EEG reports were collected. In addition, VFTs were conducted, including character VFT and semantic VFT. **B** Imaging and analysis pipeline: after acquiring T1-weighted and resting-state fMRI images, preprocessing was performed using the FSL toolbox, with further motion artifact reduction using ICA-AROMA. Group ICA was then conducted to obtain spatial templates. Functional connectivity analyses included dual regression and FSLNets-based network analysis. Finally, correlation and mediation analyses were conducted between behavioral measures and imaging metrics. Abbreviations: HCs, healthy controls; NDE, newly diagnosed epilepsy; EEG, electroencephalogram; VFT, verbal fluency test; fMRI, functional magnetic resonance imaging; ICA-AROMA, Independent Component Analysis–Automatic Removal of Motion Artifacts

All statistical analyses were performed using SPSS Statistics (version 26.0; IBM Corp) [42], and figures were generated using GraphPad Prism (version 9.0) and BioRender [43]. The workflow of this study is illustrated in Fig. 1.

## Results

### Demographic, clinical, and neurocognitive characteristics

Table 1 presents the demographic, clinical, and neurocognitive characteristics of the 100 NDE patients and 54 HCs. All participants were right-handed. The results showed no significant group differences in age, gender, or years of education (*P*s > 0.05).

**Table 1:** Demographic, clinical, and neurocognitive characteristics of the NDE and HC groups

	NDE (*N* = 100)	HCs (*N* = 54)
Demographic information		
Number of females, *n* (%)	49 (49%)	28 (52%)
Age at scanning (years, mean ± SD)	29.28 ± 11.82	27.04 ± 8.92
Years of education (mean ± SD)	12.19 ± 3.32	12.39 ± 4.75
Age of onset (years, mean ± SD)	24.47 ± 13.07	–
Clinical features		
Epilepsy duration (years, mean ± SD)	4.94 ± 6.12	–
History of febrile seizures, *n* (%)	17 (17%)	–
History of status epilepticus, *n* (%)	13 (13%)	–
Family history of epilepsy, *n* (%)	4 (4%)	–
Seizure frequency, *n* (%)		
0–1 times/year	16 (16%)	–
2–5 times/year	24 (24%)	–
6–11 times/year	27 (27%)	–
12–47 times/year	23 (23%)	–
> 47 times/year	10 (10%)	–
Seizure type, *n* (%)		
Focal onset	75 (75%)	–
Generalized onset	20 (20%)	–
Unknown onset	5 (5%)	–
Neurocognitive functions^a^		
RSPM score (mean ± SD)	48.24 ± 5.73	–
RSPM percentile (median, range)	50% (25%–97%)	–
Character VFT score (mean ± SD)	7.08 ± 2.87	–
Semantic VFT score (mean ± SD)	18.48 ± 5.40	–

*NDE* newly diagnosed epilepsy, *HCs* healthy controls, *SD* standard deviation, *VFT* verbal fluency test, *RSPM* Raven’s Standard Progressive Matrices (60 item)

^a^*N* = 84

In the NDE group, patients had a mean epilepsy duration of 4.94 years (SD = 6.12). For seizure frequency, 16% experienced 0–1 seizure per year, 24% had 2–5 seizures per year, 27% reported 6–11 seizures per year, 23% had 12–47 seizures per year, and 10% had more than 47 seizures per year. Regarding seizure types, 75% had focal-onset seizures, 20% had generalized-onset seizures, and 5% had seizures of unknown onset.

Eighty-four NDE patients completed the neurocognitive assessments. The mean RSPM score was 48.24 (SD = 5.73), with a median age-adjusted percentile of 50% (range = 25%–97%). No patients showed evidence of intellectual impairment. As verbal fluency data from HCs were not available in this study, we referred to previous reports showing that healthy adults typically produce approximately 10–15 words per minute in character VFT and 25–30 words per minute in semantic VFT [44, 45]. In comparison, our patients achieved mean scores of 7.08 (SD = 2.87) in character VFT and 18.48 (SD = 5.40) in semantic VFT.

### Brain network functional connectivity differences between groups

**Fig. 2:**
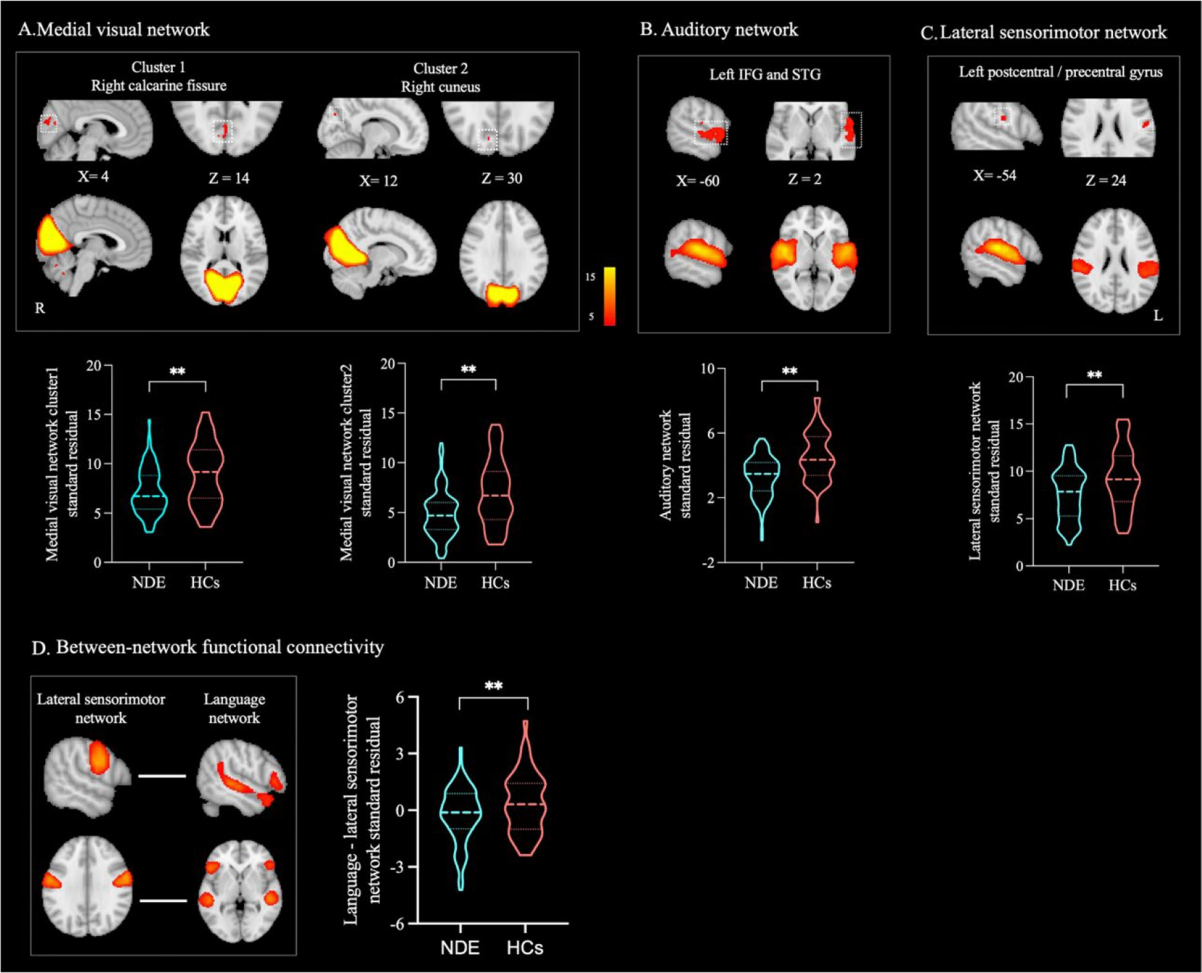
Group differences in brain network functional connectivity strengths. **A**–**C** Intra-network FC differences. Compared to HCs, the NDE group exhibited significantly reduced FC within the **A** medial visual network, **B** auditory network, and **C** lateral sensorimotor network. **D** Between-network FC differences, where the NDE group showed significantly reduced FC between the language network and the lateral sensorimotor network compared to HCs. Group differences in brain network FC are visualized in sagittal and axial views (thresholded at *z* > 5) and overlaid on the MNI152 2 mm template. The coordinates (X, Z) represent the peak voxel locations of the clusters in the MNI space. Violin plots illustrate the standardized residuals of FC *z*-scores for both NDE patients and HCs, after controlling for sex, age, and years of education as covariates. Abbreviations: IFG, inferior frontal gyrus; STG, superior temporal gyrus; FC, functional connectivity; HCs, healthy controls; NDE, newly diagnosed epilepsy. **P < 0.01

The dual regression analysis revealed that, compared to HCs, NDE patients exhibited significantly reduced intra-network FC (*P* < 0.001, FWE-corrected, cluster size ≥ 10 contiguous voxels) in the medial visual network (cluster 1: right calcarine fissure and surrounding cortex, BA17R; cluster 2: right cuneus, BA18R), auditory network (left inferior frontal gyrus [IFG], BA44L and BA45L; left superior temporal gyrus [STG], BA22L and BA41L), and lateral sensorimotor network (left postcentral gyrus and left precentral gyrus, BA3bL and BA4pL) (Fig. 2A–C). Table 2 provides details of the brain regions and peak voxel coordinates with significant group differences in intra-network FC.

**Table 2:** Group differences in intra-network functional connectivity

Brain networks	Regions	Peak voxel (MNI)				BA
		Vox	X	Y	Z	
Medial visual network						
Cluster 1	**CAL.R**	96	4	− 84	14	17R
Cluster 2	**CUN.R**	11	12	− 78	30	18R
Auditory network						
Cluster 1	**IFG.L**: STG.L	263	− 60	8	2	44/45L
Lateral sensorimotor						
Cluster 1	**PoCG.L**: PreCG.L	143	− 54	− 12	24	3b/4p L

The regions indicate the brain areas corresponding to the peak voxel in bold, while other anatomical areas within the cluster are presented in regular font. Vox refers to the number of voxels in the cluster, and X, Y, Z represent the MNI coordinates of the peak voxel within the cluster

*CAL.R *right calcarine fissure, *CUN.R *right cuneus, *IFG.L *left inferior frontal gyrus, *STG.L *left superior temporal gyrus, *PoCG.L *left postcentral gyrus, *PreCG.L *left precentral gyrus, *Vox *number of voxels, *MNI *Montreal Neurological Institute, *BA *Brodmann area, *R *right, *L *left

Building on these findings, the medial visual network, auditory network, and lateral sensorimotor network were used as ROIs to compute between-network FC (see Additional file 1: Fig. S3 for the heatmap of group comparisons for between-network FC). Finally, the FSLNets analysis showed that FC strength between the lateral sensorimotor network and language network was significantly lower in NDE patients compared to HCs (*P* < 0.05, FWE-corrected) (Fig. 2D).

### Correlation analysis results in the NDE patients

**Fig. 3:**
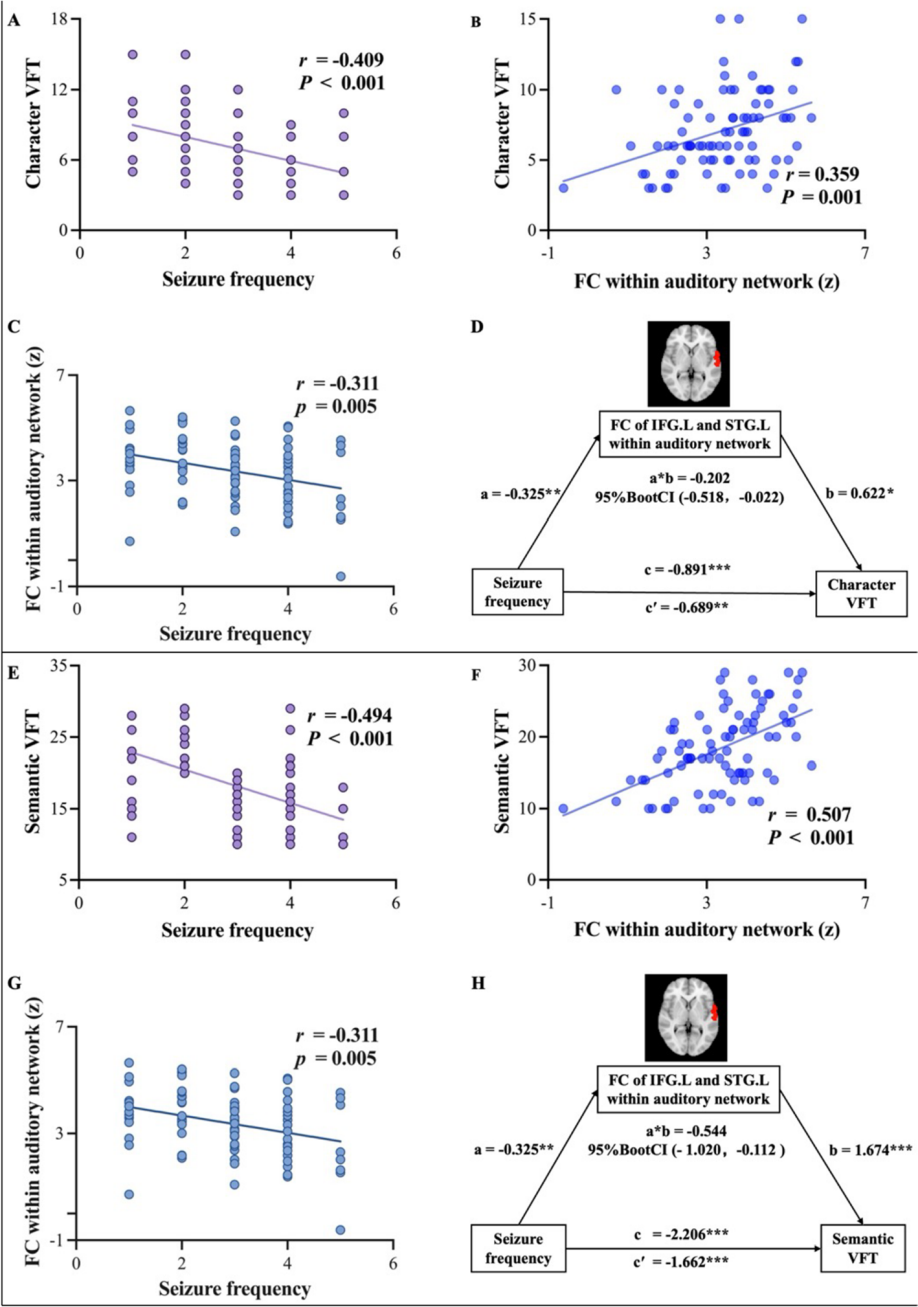
Correlation and mediation analyses of seizure frequency, VFT scores, and FC in NDE patients. **A**–**C** Correlation analyses indicate that seizure frequency is negatively correlated with character VFT scores and FC strength within the left auditory network, while FC strength within the left auditory network is positively correlated with character VFT scores. **D** Mediation analysis demonstrates that FC strength within the left auditory network mediates the relationship between seizure frequency and character VFT scores. **E**–**G** Similar correlation patterns are observed for seizure frequency, semantic VFT scores, and FC strength within the left auditory network. **H** Mediation analysis shows that FC strength within the left auditory network also mediates the relationship between seizure frequency and semantic VFT scores. Explanation of path coefficients in the mediation analyses. Path a: The direct effect of the IV on the M. Path b: The direct effect of the M on the DV. Path a*b: The indirect effect of the IV on the DV through the M. Path c′: The direct effect of the IV on the DV. Path c: The total effect of the IV on the DV, including both direct and indirect effects. 95% BootCI: The bootstrap confidence interval for the indirect effect. Abbreviations: NDE, newly diagnosed epilepsy; VFT, verbal fluency test; FC, functional connectivity; IFG.L, left inferior frontal gyrus; STG.L, left superior temporal gyrus; IV, independent variable; DV, dependent variable; M, mediator. *P < 0.05; **P < 0.01; ***P < 0.001

Correlation analyses were performed between clinical features, VFT scores, and brain network FC strengths in the NDE patients. The results showed a significant negative correlation between seizure frequency and both character VFT scores (*r* = − 0.409, *P* < 0.001) and semantic VFT scores (*r* = − 0.494, *P* < 0.001) (Fig. 3). No significant correlations were found between age of onset, epilepsy duration, and VFT scores (*P*s > 0.05).

Furthermore, intra-network FC of the left IFG and left STG within the auditory network showed significantly positive correlations with character VFT scores (*r* = 0.359, *P* = 0.001) and semantic VFT scores (*r* = 0.507, *P* < 0.001). Notably, the left IFG-STG FC also significantly negatively correlated with seizure frequency (*r* = − 0.311, *P* = 0.005) (Fig. 3). For between-network FC strengths, no significant correlations with clinical features or VFT scores were observed (*P*s > 0.05).

### Mediation analysis in the NDE patients

Mediation analysis was conducted to examine whether brain network FC strengths mediate the relationship between clinical features and verbal fluency. The results showed that intra-network FC of the left IFG and left STG within the auditory network partially mediated the effect of seizure frequency on character VFT scores (indirect effect = − 0.202, Bootstrap 95% CI = [− 0.518, − 0.022]) and semantic VFT scores (indirect effect = − 0.544, Bootstrap 95% CI = [− 1.020, − 0.112]) (Fig. 3). The mediation effect accounted for 22.67% and 24.66% of the total effect, respectively. Detailed results of the mediation analysis are provided in Additional file 2: Tables S1 and S2.

### Subgroup analysis

In the subgroup analysis of seizure type, between focal (*n* = 75) and generalized onset patients (*n* = 20), no significant differences were observed in demographics, clinical features, RSPM scores, or VFT scores (Additional file 2: Table S3). No intra- or between-network FC differences were found between the focal and generalized groups. Compared with HCs, focal onset patients exhibited reduced intra-network FC in the medial visual, auditory, and lateral sensorimotor networks (*P* < 0.001, FWE-corrected, cluster size ≥ 10 voxels) (Additional file 1: Fig. S4). No intra-network FC differences were observed between generalized onset patients and HCs.

In the subgroup analysis of laterality, 15 possible left-onset and 11 possible right-onset patients were identified, while 49 focal-onset patients could not be further lateralized due to the lack of ictal video-EEG recordings. Between the 15 possible left- and 11 possible right-onset patients, no significant differences were observed in demographics, clinical features, or RSPM scores. Possible left-onset patients performed worse on both character and semantic VFTs compared with possible right-onset patients, with the difference reaching significance for semantic VFT (*P* < 0.05, Additional file 2: Table S4). No significant intra- or between-network FC differences were observed either between the two subgroups or in comparisons with HCs.

## Discussion

**Fig. 4:**
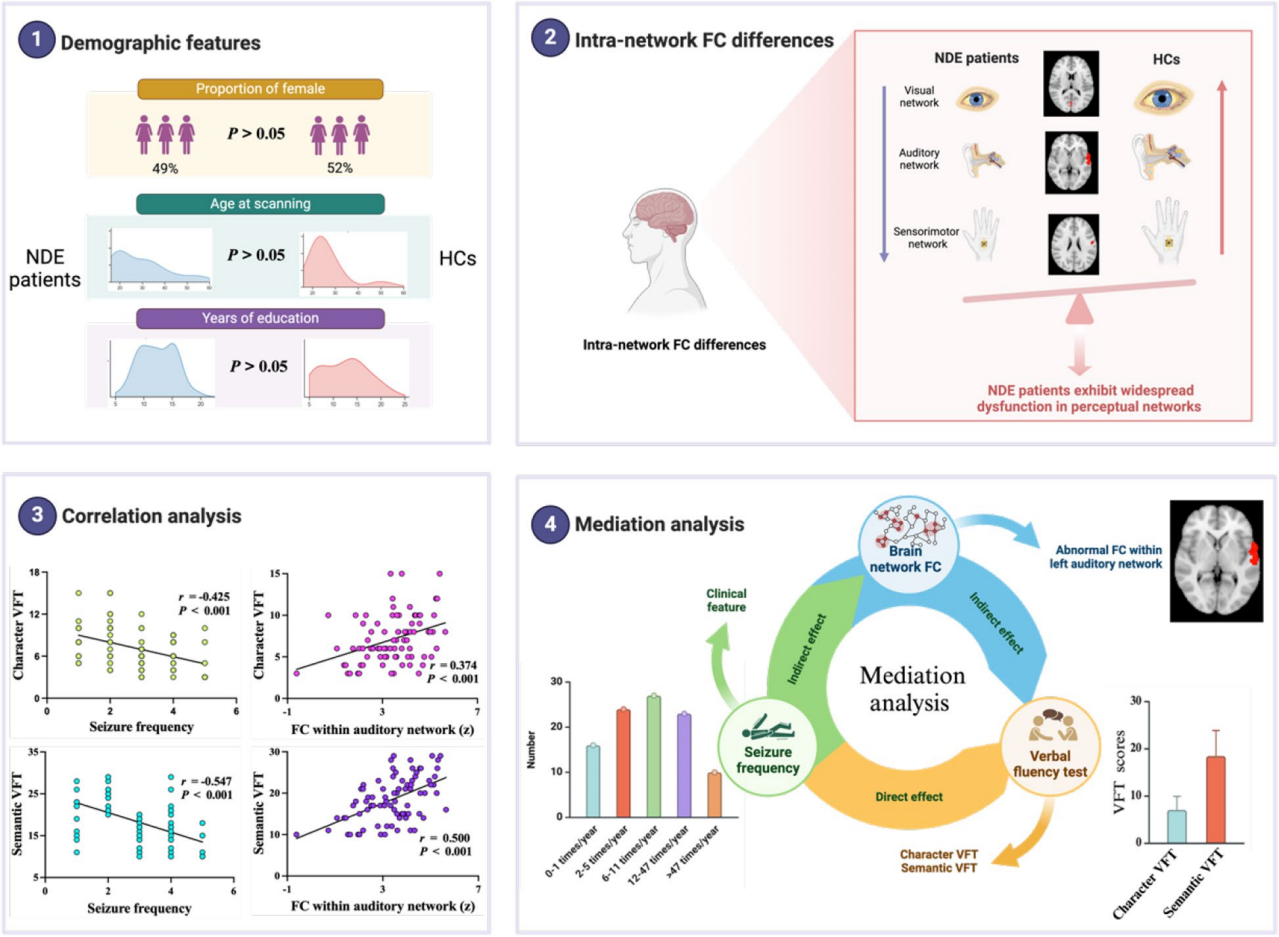
Graphical summary of key findings. NDE patients exhibited significant differences in brain network FC compared to HCs, particularly in visual, auditory, and sensorimotor networks. Disrupted auditory network FC was linked to verbal fluency impairments and mediated the relationship between seizure burden and language deficits. These findings highlight the role of auditory network dysfunction in the verbal fluency deficits observed in the NDE patients. Abbreviations: VFT, verbal fluency test; NDE, newly diagnosed epilepsy; HCs, healthy controls; FC, functional connectivity; IFG.L, left inferior frontal gyrus; STG.L, left superior temporal gyrus

This study explored resting-state whole-brain FC differences between NDE patients and HCs. Our findings showed that compared with HCs, NDE patients exhibited hypo-intra-network FC in the medial visual, auditory, and lateral sensorimotor networks. Moreover, the FC of IFG and STG within the left auditory network was significantly correlated with both character and semantic VFT scores in the NDE patients. Importantly, this FC mediated the relationship between seizure frequency and verbal fluency performance in the patients. Overall, these findings suggest that there is a widespread disruption of perceptual networks in NDE patients. Specifically, the disturbances in the IFG and STG within the left auditory network serve as the neural substrate underlying the link between seizure burden and verbal fluency deficits. A graphical summary of these findings is presented in Fig. 4.

### Brain network FC differences between the NDE patients and HCs

It is well established that epilepsy affects perception. Clinically, the most common manifestations are auras preceding seizures, such as visual auras (e.g., moving light spots), auditory auras (e.g., tinnitus), and somatosensory auras (e.g., gastric rising sensations) [46]. In addition, behavioral and electrophysiological evidence indicates that epilepsy patients exhibit multidimensional impairments in visual [47–49], auditory [50, 51], and sensorimotor functions [46, 52–54]. For example, visual evoked potential (VEP) studies have shown significantly shorter P2 wave latencies in pattern-reversal stimulation experiments in epilepsy patients compared with HCs [55]. Auditory event-related potential (ERP) studies have reported prolonged P300 latencies in epilepsy patients relative to controls [56–58]. In tactile paradigms, epilepsy patients have demonstrated poorer performance in vibratory frequency discrimination tasks [59]. However, the mechanisms underlying perceptual impairments in epilepsy remain unclear [46, 60]. Moreover, perceptual impairments have also been observed in brain regions distant from the seizure focus, suggesting that epilepsy is a network disorder affecting interconnected neural circuits [61]. Accordingly, several studies have employed intracranial EEG and high-resolution neuroimaging techniques to explore the roles of brain network alterations in the perceptual dysfunction observed in epilepsy.

Recently, noninvasive fMRI has been widely applied to epilepsy research, offering high spatial resolution for investigating large-scale network dysfunctions. Our study showed that the NDE patients exhibited hypo-intra-network FC in the medial visual, auditory, and lateral sensorimotor networks compared to HCs. Specifically, within the visual network, affected regions included the right calcarine fissure (BA17, primary visual cortex) and the right cuneus (BA18, secondary visual cortex). In the auditory network, aberrant connectivity was localized to the left IFG (BA44/45, primary regions for language production) and the left STG (BA22/41, primary auditory cortex). Additionally, the lateral sensorimotor network shows reduced FC in the left precentral gyrus (BA4) and left postcentral gyrus (BA3), which are the critical regions for motor and sensory processing. Overall, these findings indicate widespread impaired perceptual networks in the NDE patients.

Previous fMRI studies have also reported widespread alterations within perceptual networks in epilepsy; however, most have focused on chronic patient cohorts. For example, Zhang et al. observed significantly reduced FC within the auditory, sensorimotor, and the high-order visual networks in mesial temporal lobe epilepsy using ICA [62]. Li et al. reported reduced static functional connectivity (sFC) across most networks and significant reductions in dynamic functional connectivity (dFC) between the visual and sensorimotor networks in pediatric epilepsy patients [63]. Our findings in medication-naïve NDE patients show a similar pattern of perceptual network dysfunctions, suggesting that such abnormalities can already be detected at the earliest clinical stage, rather than arising solely as a consequence of chronic disease progression or long-term ASMs exposure.

At present, studies using fMRI to investigate brain network alterations in NDE patients remain limited. In 2018, Alonazi et al. conducted the first seed-based FC analysis in 27 NDE patients and reported significantly reduced connectivity between an intraparietal seed of the frontoparietal attention network and the frontal and temporal lobes [64]. As an extension, our study employed a larger sample and applied data-driven approach, to explore the whole-brain functional network alterations. Our findings further confirm that abnormal brain network changes are already present at early disease stages and additionally demonstrate widespread impaired perceptual networks.

Consistent with our findings, most studies have shown reduced FC in epilepsy patients compared with HCs, especially in newly diagnosed, suggesting the loss of connectivity within relevant brain networks are candidate causes of perceptual dysfunctions observed in epilepsy [64, 65]. In contrast, increased connectivity has been reported mainly in patients with drug resistance, or after surgery [66, 67]. These increases are usually interpreted as compensatory reorganization of brain networks.

### Verbal fluency function association with brain network FC in the NDE patients

Compared with normative data from healthy adults, NDE patients in our study showed reduced verbal fluency performance, and those with higher seizure frequency had more severe impairments. Furthermore, reduced FC within the left auditory network was correlated with the severity of verbal fluency impairment. Mediation analysis showed that the FC within the left auditory network served as a mediator in the link between the seizure frequency and the verbal fluency. These findings indicate that altered FC within the left auditory network may constitute a key neuro-mechanism underlying verbal-fluency performance deficits in the NDE patients. However, as with all cross-sectional studies, this correlation cannot be interpreted as causal, and the findings should therefore be interpreted with caution. Further longitudinal studies are needed to further confirm the causal relationships between these factors.

To better understand the role of the left auditory network in verbal fluency deficits, we considered its anatomical and functional characteristics. The left auditory network primarily consists of the STG, Heschl’s gyrus, the planum temporale, and parts of the IFG, precentral gyrus, and postcentral gyrus. This network plays a crucial role in auditory and language processing, covering functions from sound perception to language generation and comprehension [33]. Previous studies have shown that simple auditory stimuli (e.g., tones) mainly activate the primary auditory cortex (Heschl’s gyrus and planum temporale) [68, 69], while complex auditory stimuli (e.g., speech) more strongly activate the STG and superior temporal sulcus [70–73]. Additionally, processing complex auditory information involves the left IFG, which plays a key role in syntactic analysis and phonemic processing [74]. These findings indicate a functional division between the temporal and frontal lobes in language comprehension: the temporal lobe supports bottom-up auditory input and word recognition, while the frontal lobe engages in top-down regulation and integration to facilitate language understanding and expression [75]. Overall, these results support the idea that perceptual processing dysfunction may contribute to cognitive impairments [62, 76]. Previous studies have likewise shown that patients with temporal lobe epilepsy often have perceptual network impairments. When performing language tasks, their cognitive deficits may present as phonological and semantic processing difficulties [77], as well as impaired auditory and visual naming [78].

In this study, we used Chinese VFTs that are more suitable investigating language function in Chinese-speaking epilepsy patients, since the Chinese VFTs better reflect the unique linguistic characteristics of the Chinese language. Two previous task-based fMRI studies using the Chinese VFTs provide additional insights. Ci et al. found that pinyin-based VFT mainly activated the left IFG and middle temporal gyrus, while character-based VFT primarily activated the left superior frontal gyrus [20]. Similarly, Wang et al. reported that temporal lobe epilepsy patients had lower activation in the triangular and opercular parts of the left IFG and less deactivation in the anterior middle and inferior temporal gyri during character-based VFT tasks [21]. These studies indicate that Chinese versions of the VFT also primarily engages the left frontotemporal network, showing a similar activation pattern to that reported for the English version [10–13]. This suggests that core language networks may share commonalities across different language backgrounds, supporting the applicability of the Chinese VFT in Mandarin speakers; however, its relevance in non-Mandarin populations requires further validation.

### Subgroup analyses by seizure type and laterality

We further conducted subgroup analyses to explore potential differences by seizure type and laterality. Regarding seizure type, no significant differences were found between focal and generalized epilepsy patients in baseline characteristics or VFT scores, and no significant differences in intra- or between-network FC were observed between the two groups. Compared with HCs, focal epilepsy patients exhibited reduced intra-network FC within the medial visual, auditory, and lateral sensorimotor networks, consistent with the overall findings. In contrast, generalized epilepsy patients showed no significant alterations, possibly due to the relatively small sample size.

Regarding laterality, no significant differences were found in baseline characteristics between patients with possible left- and right-onset epilepsy. However, patients with possible left-onset epilepsy performed worse on VFTs, with a significant deficit in semantic VFT. This finding is consistent with previous studies, as the dominant role of the left hemisphere in language processing suggests that left-onset epilepsy may confer a higher risk of language dysfunction [79, 80]. Nevertheless, no corresponding FC differences were observed between possible left- and right-onset patients or when compared with HCs. This may be due to the limited sample size and the relatively short disease duration in NDE patients.

### Limitations

This study has several limitations. First, as a cross-sectional design, it cannot clarify the causal relationships between brain dysfunctions, clinical features, and behavioral performances, and how network abnormalities change with disease progression or ASM treatment. Longitudinal studies are needed to investigate their dynamic evolution of brain network abnormalities and its association with clinical indicators. Second, resting-state fMRI cannot precisely localize the anatomical substrates of character and semantic VFTs. Future studies incorporating task-based fMRI with language paradigms are needed to further validate our findings. Finally, all participants were recruited from a tertiary epilepsy center, which may introduce selection bias; multi-center collaborations are necessary to improve the generalizability of the results.

## Conclusions

In conclusion, our study demonstrates that widespread dysfunction of perceptual networks is evident in early epilepsy, rather than developing progressively with disease course or ASM exposure. Abnormal FC within the left auditory network, particularly in the IFG and STG, may represent a neural substrate for impaired verbal fluency in the NDE patients. These findings provide potential precise targets for neuromodulation strategies that could improve cognitive outcomes and long-term prognosis in patients with epilepsy.

## Supplementary Information

Additional file 1. Figures S1–S4. Fig. S1 Pipeline for preprocessing of resting-state fMRI data. Fig. S2 The 18 intrinsic connectivity networks. Fig. S3 Heatmap of between-network functional connectivity differences. Fig. S4 Intra-network functional connectivity differences within subgroup analyses of seizure types.

Additional file 2. Tables S1–S4. Table S1 The mediation effect of intra-network FC of the left auditory network on the relationship between seizure frequency and character VFT scores. Table S2 The mediation effect of intra-network FC of the left auditory network on the relationship between seizure frequency and semantic VFT scores. Table S3 Demographic, clinical, and neurocognitive characteristics of the focal onset, generalized onset, and HCs groups. Table S4 Demographic, clinical, and neurocognitive characteristics of the possible left onset, possible right onset, and HCs groups.

## Data Availability

The data will be available with a reasonable request.

## Abbreviations

ASMs: Anti-seizure medications
fMRI: Functional magnetic resonance imaging
FC: Functional connectivity
HCs: Healthy controls
IFG: Inferior frontal gyrus
ICA: Independent component analysis
ICNs: Intrinsic connectivity networks
NDE: Newly diagnosed epilepsy
ROIs: Regions of interest
STG: Superior temporal gyrus
VFTs: Verbal fluency tests
