# Supplementary material for: Network dysfunction underlying verbal fluency deficits in newly diagnosed epilepsy: a resting-state fMRI functional connectivity study
Source: BMC Med. 2025 Dec 13;24:35. doi: 10.1186/s12916-025-04577-y (PMC12817765; doi:10.1186/s12916-025-04577-y)
Supplement: Supplementary file 1 — Additional file 1. Figures S1–S4. Fig. S1 Pipeline for preprocessing of resting-state fMRI data. Fig. S2 The 18 intrinsic connectivity networks. Fig. S3 Heatmap of between-network functional connectivity differences. Fig. S4 Intra-network functional connectivity differences within subgroup analyses of seizure types. [file 12916_2025_4577_MOESM1_ESM.docx]

#### ****Fig. S1****


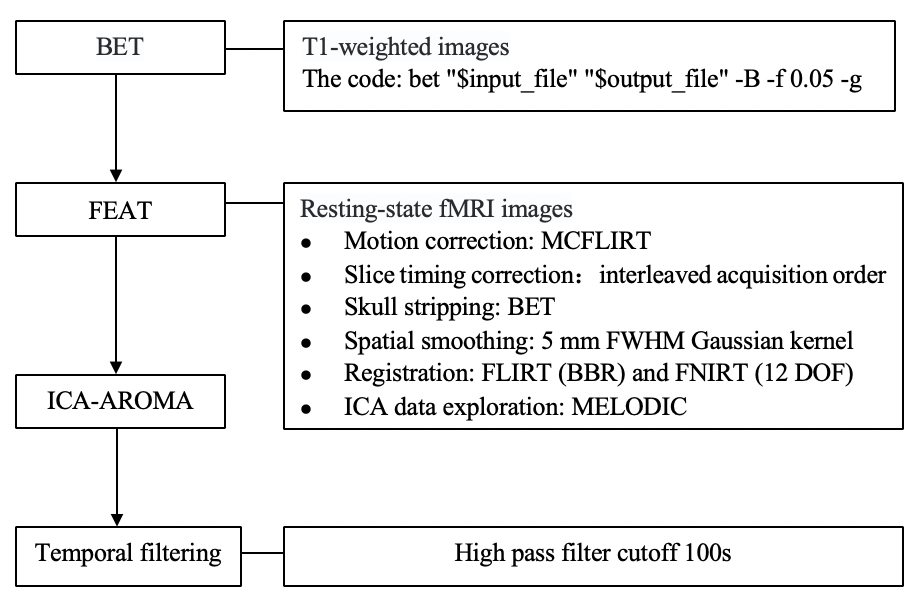


**Fig. S1. Pipeline for preprocessing of resting-state fMRI data.** The flowchart illustrates the main steps of image preprocessing. T1-weighted structural images were skull-stripped using BET (Brain Extraction Tool) and used for functional MRI (fMRI) registration. Preprocessing of resting-state fMRI data included motion correction (MCFLIRT), slice-timing correction (interleaved acquisition order), skull stripping, spatial smoothing with a 5-mm FWHM Gaussian kernel, and registration using FLIRT (BBR) and FNIRT (12 DOF). Independent component analysis (ICA) was performed with MELODIC to identify individual functional networks. ICA-AROMA (Automatic Removal of Motion Artifacts) was then applied to further reduce motion-related noise. Following the official ICA-AROMA guidelines, temporal filtering (high-pass filter cutoff = 100 s) was conducted after ICA-AROMA.

**Abbreviations:** BET, Brain Extraction Tool; FEAT, FMRI Expert Analysis Tool; MCFLIRT, Motion Correction FMRIB’s Linear Image Registration Tool; FWHM, full width at half maximum; BBR, Boundary-Based Registration; DOF, degrees of freedom; FLIRT, FMRIB’s Linear Image Registration Tool; FNIRT, FMRIB’s Nonlinear Image Registration Tool; ICA-AROMA, Independent Component Analysis–Automatic Removal of Motion Artifacts.

#### ****Fig. S2****


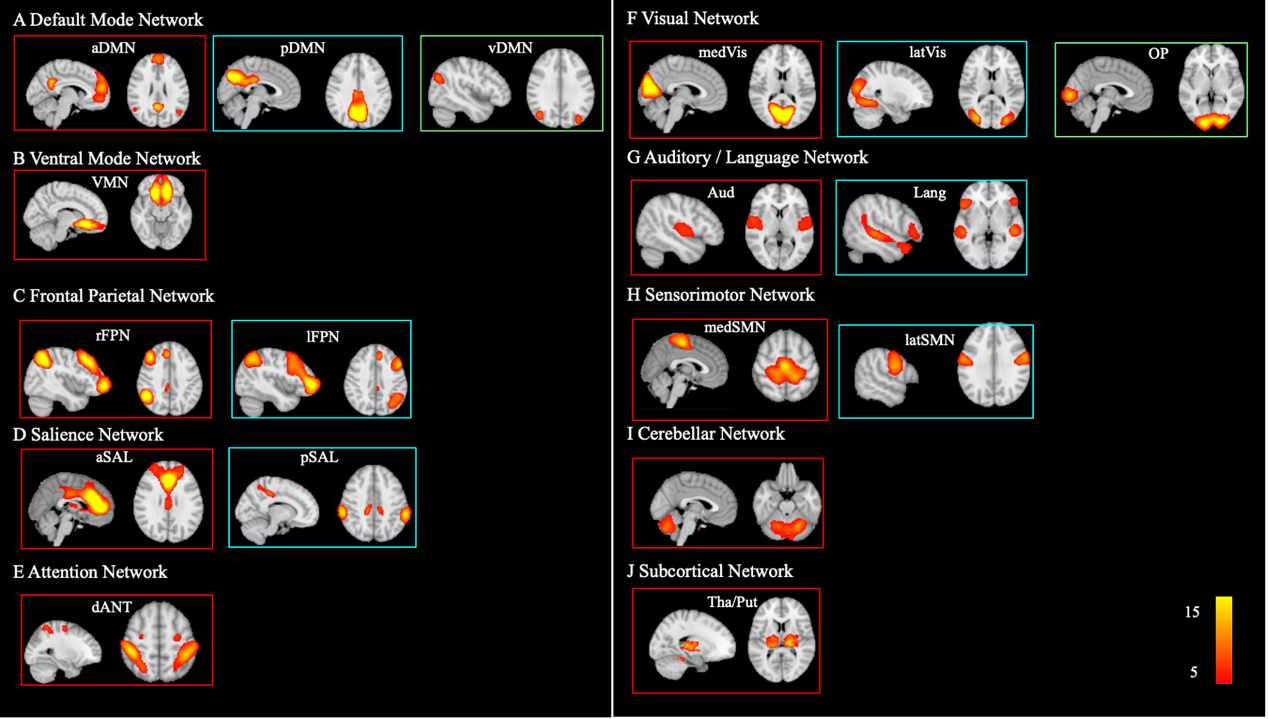


**Fig. S2. The 18 intrinsic connectivity networks.** The figure illustrates 18 resting-state brain networks identified through group-level independent component analysis (threshold: z > 5). Spatial maps are shown in sagittal and axial views, overlaid on the MNI152 2mm standard space template.

**Abbreviations:** DMN, default mode network; a, anterior; p, posterior; v, ventral; VMN, ventral mode network; FPN, frontoparietal network; r, right; l, left; SAL, salience network; dANT, dorsal attention network; Vis, visual network; med, medial; lat, lateral; OP, occipital pole; Aud, auditory network; Lang, language network; SMN, sensorimotor network; Tha, thalamus; Put, putamen.

#### ****Fig. S3****


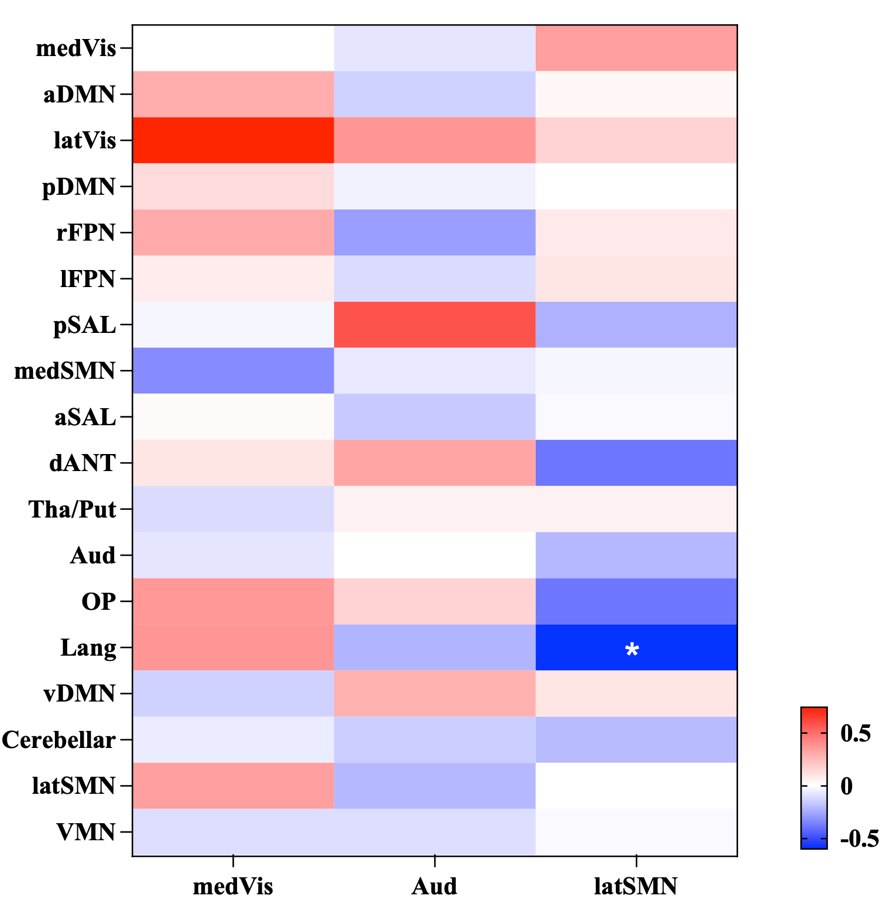


**Fig. S3. Heatmap of between-network functional connectivity (FC) differences.** The figure displays group differences in FC between three seed networks and 18 intrinsic connectivity networks. Colors represent Fisher r-to-z–transformed correlation coefficients (z-scores). Warmer colors indicate higher FC in newly diagnosed epilepsy (NDE) patients compared with healthy controls (HCs), whereas cooler colors indicate lower FC in NDE relative to HCs. Significant between-group differences are marked with an asterisk (*, *P* < 0.05, FWE-corrected).

**Abbreviations:** DMN, default mode network; a, anterior; p, posterior; v, ventral; VMN, ventral mode network; FPN, frontoparietal network; r, right; l, left; SAL, salience network; dANT, dorsal attention network; Vis, visual network; med, medial; lat, lateral; OP, occipital pole; Aud, auditory network; Lang, language network; SMN, sensorimotor network; Tha, thalamus; Put, putamen.

#### ****Fig. S4****

**
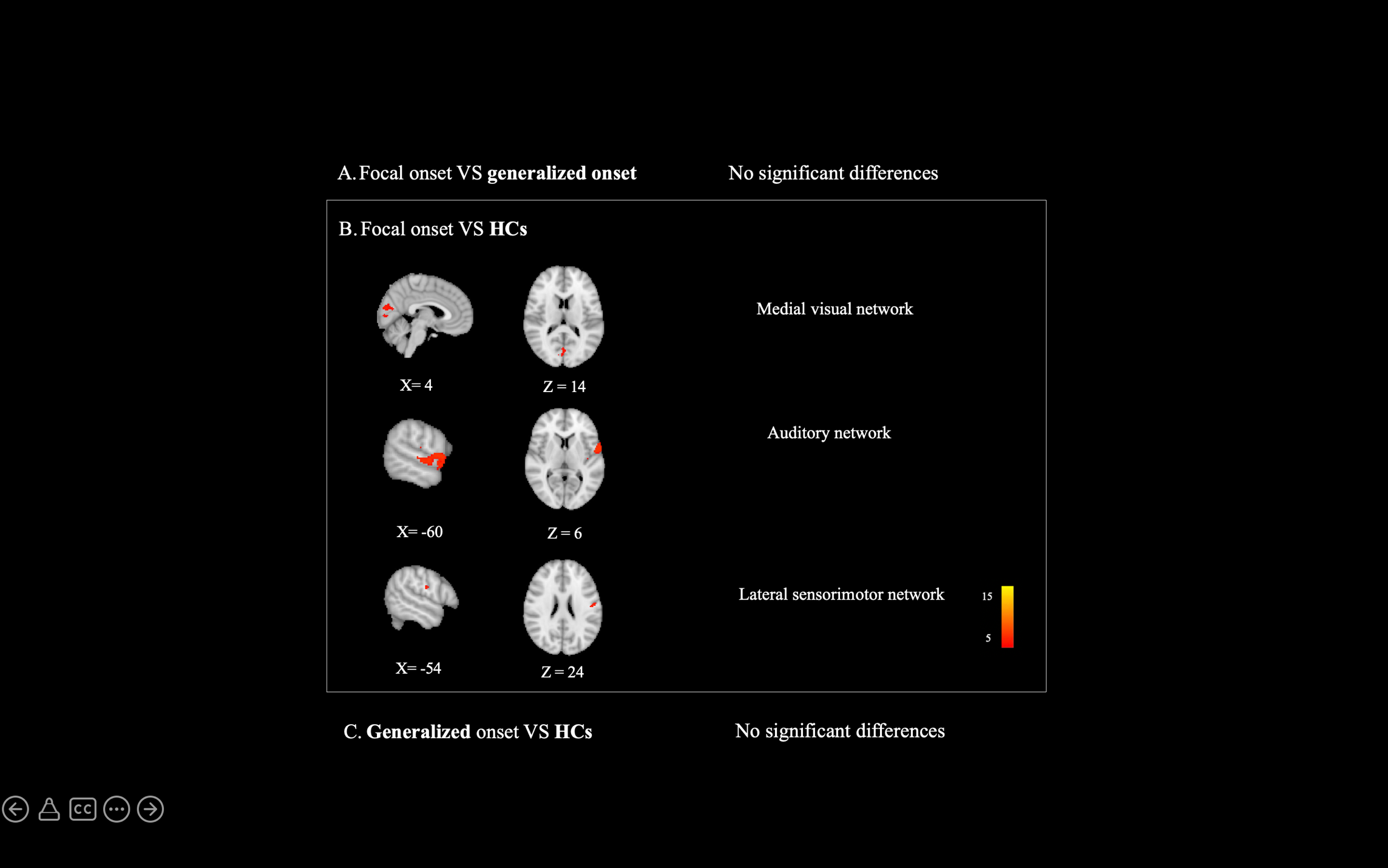
Fig. S4. Intra-network functional connectivity (FC)** **differences within subgroup analyses of seizure types. (A) No significant differences were found between focal onset and generalized onset patients. (B) Compared with** healthy controls (HCs)**, focal onset patients exhibited significantly reduced intra-network FC in the medial visual, auditory, and lateral sensorimotor networks (*P* < 0.001, FWE corrected, cluster size ≥ 10 voxels). (C) No significant differences were observed between generalized onset patients and HCs.** Group differences in brain network FC are visualized in sagittal and axial views (threshold: z > 5) and overlaid on the MNI152 2mm template. The coordinates (X, Z) represent the peak voxel locations of the clusters in MNI space.
