# Supplementary material for: Network dysfunction underlying verbal fluency deficits in newly diagnosed epilepsy: a resting-state fMRI functional connectivity study
Source: BMC Med. 2025 Dec 13;24:35. doi: 10.1186/s12916-025-04577-y (PMC12817765; doi:10.1186/s12916-025-04577-y)
Supplement: Supplementary file 2 — Additional file 2. Tables S1–S4. Table S1 The mediation effect of intra-network FC of the left auditory network on the relationship between seizure frequency and character VFT scores. Table S2 The mediation effect of intra-network FC of the left auditory network on the relationship between seizure frequency and semantic VFT scores. Table S3 Demographic, clinical, and neurocognitive characteristics of the focal onset, generalized onset, and HCs groups. Table S4 Demographic, clinical, and neurocognitive characteristics of the possible left onset, possible right onset, and HCs groups. [file 12916_2025_4577_MOESM2_ESM.docx]

#### ****Table S1.** The mediation effect of intra-network FC of the left auditory network on the relationship between seizure frequency and character VFT scores.**

| Effects | Coefficients | SE | Bootstrap 95% CI | | Coefficient ratio |
| --- | --- | --- | --- | --- | --- |
|  |  |  | Lower threshold | Upper threshold |  |
| Total effects | -0.891 | 0.253 | -1.394 | -0.387 |  |
| Direct effects | -0.689 | 0.258 | -1.201 | -0.176 | 77.329% |
| Indirect effects | -0.202 | 0.130 | -0.518 | -0.022 | 22.671% |

**Abbreviations:** FC, functional connectivity; VFT, verbal fluency test; SE: standard error; CI, confidence interval.

#### ****Table S2.** The mediation effect of intra-network FC of the left auditory network on the relationship between seizure frequency and semantic VFT scores.**

| Effects | Coefficients | SE | Bootstrap 95% CI | | Coefficient ratio |
| --- | --- | --- | --- | --- | --- |
|  |  |  | Lower threshold | Upper threshold |  |
| Total effects | -2.206 | 0.440 | -3.081 | -1.331 |  |
| Direct effects | -1.662 | 0.421 | -2.500 | -0.824 | 75.340% |
| Indirect effects | -0.544 | 0.235 | -1.020 | -0.112 | 24.660% |

**Abbreviations:** FC, functional connectivity; VFT, verbal fluency test; SE: standard error; CI, confidence interval.

#### ****Table S3****. Demographic, clinical, and neurocognitive characteristics of the focal onset, generalized onset and HCs groups

|  | Focal (N=75) | Generalized (N=20) | HCs (N=54) |
| --- | --- | --- | --- |
| **Demographic information** |  |  |  |
| Number of females, n (%) | 36 (48%) | 11(55%) | 28 (52%) |
| Age at scanning (years, mean ± SD) | 29.71 ± 12.43 | 27.40 ± 10.96 | 27.04 ± 8.92 |
| Years of education (mean ± SD) | 12.32 ± 3.34 | 11.80 ± 3.27 | 12.39 ± 4.75 |
| Age of onset (years, mean ± SD) | 25.16 ± 13.70 | 23.40 ± 11.98 | – |
|  |  |  |  |
| **Clinical features** |  |  |  |
| Epilepsy duration (years, mean ± SD) | 4.72 ± 5.94 | 4.04 ± 4.78 | – |
| History of febrile seizures, n (%) | 13 (17%) | 3 (15%) | – |
| History of status epilepticus, n (%) | 10 (13%) | 2 (10%) | – |
| Family history of epilepsy, n (%) | 3 (4%) | 1 (5%) | – |
|  |  |  |  |
| Seizure frequency, n (%) |  |  |  |
| 0-1 times/year | 10 (13%) | 6 (30%) | – |
| 2-5 times/year | 18 (24%) | 5 (25%) | – |
| 6-11 times/year | 20 (27%) | 5 (25%) | – |
| 12-47 times/year | 19 (25%) | 3 (15%) | – |
| >47 times/year | 8 (11%) | 1 (5%) | – |
|  |  |  |  |
| **Neurocognitive functions^a^** |  |  |  |
| RSPM score (mean ± SD) | 48.46 ± 6.08 | 47.61 ± 4.45 | – |
| RSPM percentile (median, range) | 55% (25%-97%) | 50% (25%-90%) | – |
| Character VFT score (mean ± SD) | 7.06 ± 2.90 | 7.00 ± 2.83 | – |
| Semantic VFT score (mean ± SD) | 18.26 ± 5.61 | 19.50 ± 4.58 | – |

**Abbreviations:** NDE, newly diagnosed epilepsy; HCs, healthy controls; SD, standard deviation; VFT, verbal fluency test; RSPM, Raven’s Standard Progressive Matrices (60 item).

a*N* = 83.

#### ****Table S4.**** Demographic, clinical, and neurocognitive characteristics of the possible left onset, possible right onset, and HCs groups

|  | Possible left (N=15) | Possible right (N=11) | HCs (N=54) |
| --- | --- | --- | --- |
| **Demographic information** |  |  |  |
| Number of females, n (%) | 10 (67%) | 5 (45%) | 28 (52%) |
| Age at scanning (years, mean ± SD) | 27.87 ± 12.06 | 32.27 ± 14.66 | 27.04 ± 8.92 |
| Years of education (mean ± SD) | 13.00 ± 3.38 | 11.91 ± 3.91 | 12.39 ± 4.75 |
| Age of onset (years, mean ± SD) | 24.80 ± 13.41 | 28.00 ± 14.81 | – |
|  |  |  |  |
| **Clinical features** |  |  |  |
| Epilepsy duration (years, mean ± SD) | 3.22 ± 4.98 | 4.32 ± 3.98 | – |
| History of febrile seizures, n (%) | 1 (7%) | 2 (18%) | – |
| History of status epilepticus, n (%) | 2 (13%) | 1 (9%) | – |
| Family history of epilepsy, n (%) | 0 (0%) | 0 (0%) | – |
|  |  |  |  |
| Seizure frequency, n (%) |  |  |  |
| 0-1 times/year | 2 (13%) | 1 (30%) | – |
| 2-5 times/year | 7 (47%) | 5 (25%) | – |
| 6-11 times/year | 3 (20%) | 2 (25%) | – |
| 12-47 times/year | 2 (13%) | 2 (15%) | – |
| >47 times/year | 1 (7%) | 1 (5%) | – |
|  |  |  |  |
| **Neurocognitive functions^a^** |  |  |  |
| RSPM score (mean ± SD) | 49.92 ± 6.19 | 48.73 ± 4.05 | – |
| RSPM percentile (median, range) | 75% (30%-97%) | 65% (35%-85%) | – |
| Character VFT score (mean ± SD) | 6.18 ± 2.18 | 8.69 ± 3.09 | – |
| Semantic VFT score (mean ± SD) * | 18.82 ± 4.60 | 19.15 ± 6.78 | – |

**Abbreviations:** NDE, newly diagnosed epilepsy; HCs, healthy controls; SD, standard deviation; VFT, verbal fluency test; RSPM, Raven’s Standard Progressive Matrices (60 item).

a*N* = 25. ****P* < 0.05.**
